# The Association between Socioeconomic Characteristics and Consumption of Food Items among Brazilian Industry Workers

**DOI:** 10.1100/2012/808245

**Published:** 2012-05-22

**Authors:** Daniele B. Vinholes, Ione M. F. Melo, Carlos Alberto Machado, Hilton de Castro Chaves, Flavio D. Fuchs, Sandra C. Fuchs

**Affiliations:** ^1^Postgraduate Studies Program in Epidemiology, School of Medicine, Universidade Federal do Rio Grande do Sul, Rua Ramiro Barcelos 2600, CEP 90035-003 Porto Alegre, RS, Brazil; ^2^Unit of Health and Safety of Work, Serviço Social da Indústria (SESI), SBN - Quadra 01 - Bloco C - Ed. Roberto Simonsen, CEP 70040-903 Brasília, DF, Brazil; ^3^Coordenação Nacional de Hipertensão e Diabetes do Departamento de Atenção Básica, Ministério da Saúde, SAF/Sul, Trecho 02, Lote 05/06 - Torre II, CEP 70070-600 Brasília, DF, Brazil; ^4^Disciplina de Cardiologia, Escola Paulista de Medicina, UNIFESP, Pedro de Toledo, 720 - Vila Mariana, CEP 04039-002 São Paulo, SP, Brazil; ^5^Departamento de Medicina Clínica, Universidade Federal de Pernambuco, Av. Prof. Moraes Rego, 1235-Cidade Universitária, CEP 50670-901 Recife, PE, Brazil; ^6^Postgraduate Studies Program in Cardiology, School of Medicine, Universidade Federal do Rio Grande do Sul, Rua Ramiro Barcelos 2600, CEP 90035-003 Porto Alegre, RS, Brazil

## Abstract

*Background*. Dietary pattern plays a causative role in the rising of noncommunicable diseases. The SESI (*Serviço Social da Indústria*) study was designed to evaluate risk factors for noncommunicable diseases. We aimed to describe food items consumed by Brazilian workers and to assess their association with socioeconomic status. *Methods*. Cross-sectional study was carried out among Brazilian industrial workers, selected by multistage sampling, from 157 companies. Interviews were conducted at the work place using standardized forms. *Results*. 4818 workers were interviewed, aged 35.4 ± 10.7 years, 76.5% were men. The workers had an average of 8.7 ± 4.1 years of schooling and 25.4 ± 4.1 kg/m^2^ of BMI. Men and individuals with less than high school education were less likely to consume dairy products, fruits, and vegetables daily, even after control for confounding factors. Men consumed rice and beans daily more often than women. In comparison to workers aged 50–76 years, those under 30 years old consumed less fruits and green leafy vegetables daily. *Conclusion*. The food items consumed by Brazilian workers show that there are insufficient consumption according to the guidelines of healthy foods, particularly of dairy products, vegetables, and fruits.

## 1. Background

The dietary pattern has a causality role in the rising of noncommunicable diseases, particularly of cardiovascular diseases. Hypertension [1], diabetes mellitus [2], and coronary heart disease [3] are major examples of such consequences. In order to identify dietary habits associated with the risk of disease is at first necessary to assess the intake of dietary components in order to derive dietary patterns and evaluate their associations with health conditions or diseases. Analyses of food intake to derive dietary patterns have been proposed by several investigators [4, 5]. The description of an overall dietary pattern is appealing since it may reproduce the way that people eat and may be used to estimate the risk of individuals [6] and to establish a linkage to biological, behavioral, and socioeconomic characteristics.

Higher socioeconomic levels are associated with healthier choice of foods [7–9]. Subjects with high income and educational level tend to have access and resources to consume fruits, vegetables [10, 11], and dairy products [12, 13]. Among women, dietary choices are affected by socioeconomic factors, including, but not exclusively, the food prices [8]. In the absence of major economic restriction, dietary choices are usually based on individual preferences, [13] which are more likely to be modified by lifestyle interventions [14]. Workers constitute a subgroup of people with enough income to provide their nutritional demands and healthy enough to work. The Brazilian workers have a large range of salaries and, as a complement, they receive food stamps or are beneficiaries of well-balanced diets at the work place. Therefore, even those with low income and educational level may have access to more appropriate diets. In Brazil, the association between dietary pattern and socioeconomic level has been investigated and the results pointed out for a direct association between socioeconomic level and healthy dietary patterns [8, 9].

Strategies to introduce healthier habits among workers aim to prevent the incidence of noncommunicable diseases, to lower the rates of absenteeism, to increase productivity and to reduce healthcare expenditures [15–17]. These strategies are integrated by public health policies to improve nutritional status of workers through the Worker Food Programe (*Programa de Alimentação do Trabalhador*), regulated in 1976, in order to provide food to low wage workers. The program has financial support of the Brazilian government, and is available to employees and employers [18].

The SESI *(Industry Social Work, Serviço Social da Indústria) *study, was conducted among the Brazilian industry workers, between 2006 and 2008, and it was the first nationwide educational intervention designed to modify risk factors for noncommunicable diseases at the work place. The first phase of this study comprised the execution of a cross-sectional survey in a representative sample of the Brazilian workers to identify their cardiovascular risk profile and eating patterns. The results of this study were used to evaluate the subsequent intervention. In this paper, we described the consumption of food items and assessed the association of daily intake with socioeconomic characteristics of Brazilian industry workers.

## 2. Material and Methods

### 2.1. Design and Population

Cross-sectional study was carried out among the Brazilian workers, randomly selected by multistage sampling, from 157 companies registered in the Annual Listing of Social Information (RAIS), of the Ministry of Labor and Employment. The RAIS is a nationwide central registry including all Brazilian companies, regularly updated by regulatory agencies of each state. Brazilian workers aged 15 years or older were selected from stratums of small (20–99 employees), medium (100–499 employees), and large (≥500 employees) companies in each state. 

### 2.2. Sampling and Sample Size Calculation

Workers were randomly selected by multistage sampling. The first stage was a stratum of with regions of the country, built with one state per region (*Alagoas, Mato Grosso do Sul, Tocantins, Rio de Janeiro and Rio Grande do Sul*), selected through simple random sampling. The second stage was the size of the company, composed by small, medium, and large companies listed in the RAIS, in 2003. A stratified random sample, proportional to the stratum size, was used to establish the number of small, medium and large companies per state, and accordingly the number of workers. Companies, ordered by city, were thereafter selected by systematic random sampling at each stratum. Prior to data collection, the companies were visited by the supervisors in order to inform managers about the project, to obtain consent from the participating company, and to verify the structure of the company to generate the systematic random sample of workers. In each company, a random systematic sampling was used to select workers. All sampling procedures were developed in details before the data collection start by an epidemiologist and a statistician, and during the field work two supervisors reassure that the steps have been followed. 

Since there was no data related to the prevalence of risk factors among industry workers, the sample size calculation was based on theoretical prevalence of cardiovascular risk factors ranging between 5% and 50%, with a sampling error of 5%. The sample size adequate for tests of association was based on the assumption that 5000 men and women would be necessary to detect a prevalence ratio of at least 1.2, with 80% power and 5% significance level (two-tailed) for a prevalence of risk factors of 12% among unexposed and 21% among exposed, with a 1 : 1 ratio, and accounting for losses. This sample was drawn to be representative of a total of 5.453.439 workers registered in RAIS. Epi Info 2000 (Centers for Disease Control and Prevention, Atlanta, USA), version 3.3.2, was used for sample size calculation. 

### 2.3. Studied Variables

Participants were interviewed at the work place using a standardized questionnaire, which included assessment of demographic (sex and age), socioeconomic (education-years of schooling), and size of the company (small, medium, and large), and other characteristics. Years of schooling were used as a marker for socioeconomic be strongly associated with income. 

Age was calculated subtracting birth from interview date, and categorized into 15–29, 30–39, 40–49, and 50 years or older. Years of formal education were categorized into 0–4, 5–8, 9–11, and 12 or over. The size of company (small, medium and large) was those established by the regulatory agencies regarding the number of employees (small: 20–99, medium: 100–499, and large: ≥500). 

Food items intake was assessed using a food-frequency questionnaire (FFQ), which included questions about the consumption of unhealthy food—high content of sodium, or fat—and healthy food, such as dairy products, vegetables, and fruits—high content of potassium, calcium, and fiber—based on the current guidelines to prevent hypertension and cardiovascular diseases by the Brazilian Societies of Cardiology, Hypertension, and Nephrology (IV Brazilian Guidelines on Hypertension) [19]. The FFQ has been extensively tested in companies not selected for study, in order to verify whether the wording was appropriate to the workers of each state. The pilot study was carried out one state, Rio Grande do Sul, and enrolled 291 workers. During the analysis, food items were presented according food groups: dairy products, cereals products, fruits and vegetables, beans, meats, oils and fats, fast food, and sweets. The answers of the FFQ had five options of answer, ranging from daily to never or rarely. Food items were categorized into daily, 4 to 6 times per week, 2 to 3 times per week, 1 time per week, and less than 1 time per week. 

Weight (kg) was measured (with subject in light clothing and barefoot) to the nearest 100 g with an electronic scale (Plenna, model Mea—07400), and height (cm) was measured with barefoot participants positioned with heels, buttocks, back and head against the wall, and the head aligned in the Frankfort horizontal plane, to the nearest 0.1 cm using a stadiometer (Tonelli, vertical model). The head is in the Frankfort plane when the horizontal line from the ear canal to the lower border of the orbit of the eye is parallel to the floor and perpendicular to the vertical wall [20]. Body mass index (BMI = weight (kg)/height (m^2^)) was calculated. Obesity was defined by a BMI ≥30 kg/m^2^. 

### 2.4. Research Team and Quality Control

The research protocol was pretested in a pilot study carried out in Canoas, Rio Grande do Sul, among 291 workers (210 men and 81 women). The analysis of the pilot study resulted in the adjustment of the questionnaire and other instruments. 

In each participating state, a research team was formed including coordinators, supervisors of work field, and interviewers. The research teams were trained to perform the interviews, anthropometric and blood pressure measurements. The mistakes were corrected and the protocol reinforced. A total of 148 professionals (undergraduate students, technicians, and nurses) were certified using a standardized protocol. New training sessions were conducted in the middle term to reassure quality of data gathering. The teams received support from the National Department of SESI for the fieldwork planning, besides training and supervision. The interviews and measurements were codified and reviewed by the state coordinators, and 10% of questionnaires were sent back to clarify or obtain additional information. The data was entered in duplicate to reduce the typing errors. The Institutional Review Board and the Ethics Committee of the University Federal do Rio Grande do Sul approved the protocol, and all participants provided informed consent. 

### 2.5. Statistical Analysis and Data Presentation

Calculation of the sample size was based on an estimate of the prevalence of daily intake of whole milk by women (50%) and by men (40%), in order to assure a power not lower than 80%, with a significance level of 0.05 (two sided). Assuming that the ratio between exposed and non-exposed groups was 3 : 1, 1080 workers were needed. Since the SESI study had other objectives, the sample size was increased. 

The descriptive analysis was presented using percentages, and multidimensional graphical format focused on the outer limits of dietary intake stratified by sex. In the Figures, the consumption of food items was indicated by a reference circle of radius ranging from 0 to 100%. Differences between categories of food item consumption by sex were tested by Pearson chi-square in the bivariate analysis, and an analysis that takes into account of the ordering for frequency of consumption, the multinomial regression, adjusted for age, years at school, and size of the company. Since differences in consumption were efficiently delivered by percentages, the *P* values of the multinomial regression analysis using were presented, showing the statistically significances. All analyses were weighted by the sampling effect. The data analysis was conducted using the Statistical Package for Social Sciences, version 16.0, Chicago, IL, USA. 

## 3. Results

In the total, 4818 workers were interviewed, aged 35.4 ± 10.7 years, 76.5% were men, had on average 8.7 ± 4.1 years at school, and 12% were obese.  Table 1 shows characteristics of the Brazilian workers. The response rate was 96% for individuals. Participants worked at 157 companies (5 in Alagoas, 11 in Mato Grosso do Sul, 63 in Rio de Janeiro, 73 in Rio Grande do Sul, and 5 in Tocantins) of small (*n* = 105), medium (*n* = 35), and large (*n* = 17) size, and the response rate was 93% for the companies. The main reasons for refusals were the time needed to conducted the interview and to deliver the educational intervention implemented in the work place, companies that were on collective vacation, and losses due to companies that went out of business. 

**Figure 1:**
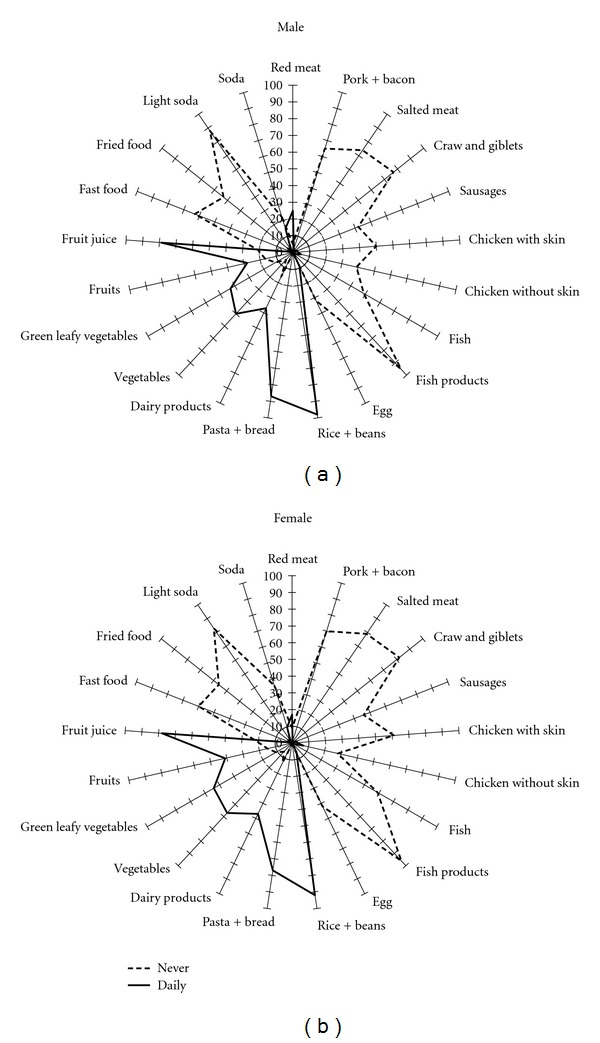
Food frequency consumed daily and never according to sex, SESI study.

Figure 1 shows the frequency of food items daily and never or rarely consumed among men and women. The shape of the distribution of food items consumed was similar among men and women, but the area of the graphic was greater for women than men. Daily food intake was dominated by rice and bean, pasta and bread, and fruit juice among men and women. On the other side, light soda, and fish products were rarely consumed by workers. Daily intake of vegetables (*P* < 0.001), leafy vegetables (*P* < 0.001), dairy products (*P* < 0.001), and fruits (*P* < 0.001) were more frequent among women; while red meat (*P* < 0.001), rice and beans (*P* < 0.001), and pasta and bread (*P* < 0.001) were more frequent among men. Pork and bacon, salted meat, craw and giblets, and fish products were rarely consumed by more than 60% of workers. On the other side, skim milk was never consumed by barely 90% of workers and almost 50% never drink whole milk. 

**Figure 2:**
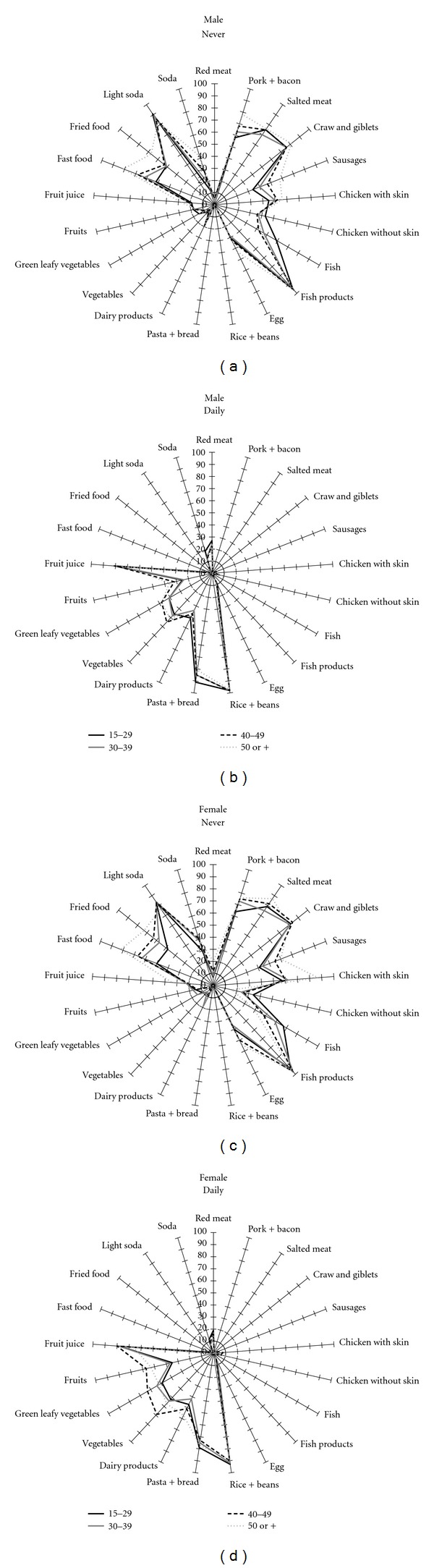
Food frequency consumed daily and never according to age, stratified by sex, SESI study.

**Figure 3:**
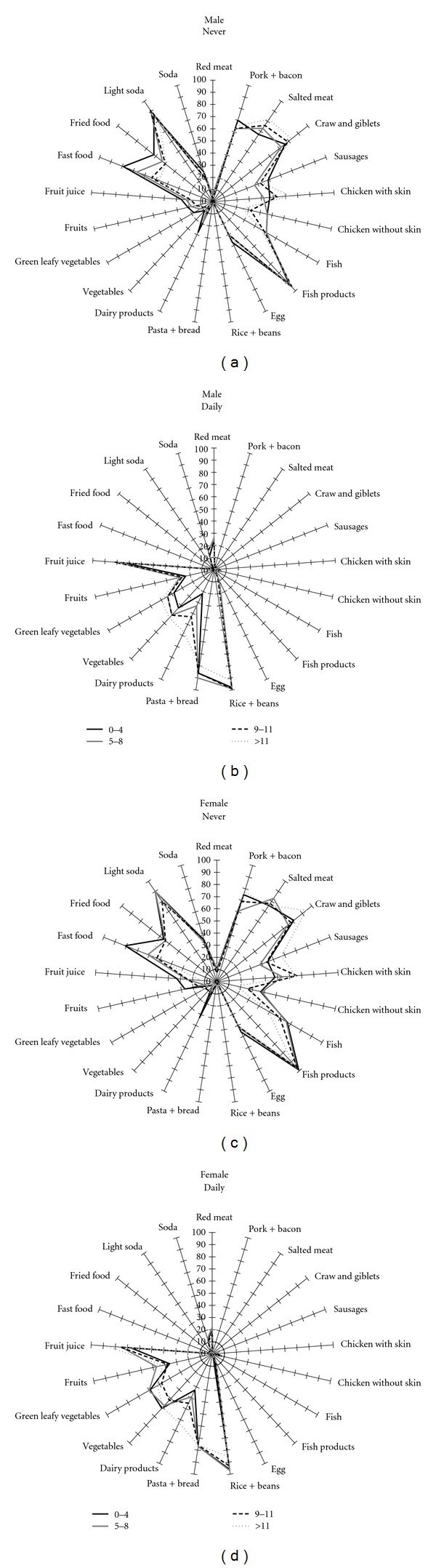
Food frequency consumed daily and never according to education, stratified by sex, SESI study.

Figures 2 and 3 show extreme categories of dietary intake by age and education level, stratified by sex.  Figure 2 shows that for men and women the daily intake of fruits (*P* < 0.001), vegetables (*P* < 0.001), and green leafy vegetables (*P* = 0.001 and *P* = 0.007, resp.) tended to increase with age. There was a reduction of daily intake of pasta and bread with age among men (*P* < 0.001), but not in women. Daily consumption of dairy products had an inverse association with age among men (*P* = 0.009), but there was a trend toward increase with ageing in women (*P* = 0.08). Fish, chicken with or without skin, pork or bacon, fast food and fried food were the food items less often consumed by the workers and the low consumption varied according to the age category. The daily consumption varied with schooling (Figure 3). 

Figure 3 shows that the daily consumption varied according to schooling. Red meat, for instance, increased from 24% to 28% between male workers with low (0–4 years) and high (>11 years) education level (*P* = 0.04), but among women, the consumption reduced from 20% to 12% (*P* = 0.005) for the same categories of schooling. Rice and beans, on the other side, have been less consumed by more educated workers (*P* < 0.001), while dairy products have increased (*P* < 0.001). Male workers with high school education or overconsumed less pasta and bread daily than those with incomplete elementary school, as well as rice and beans among female workers. 

Table 2 describes the frequency of food consumption according to sex. Data about absolute numbers and percentages were presented. Furthermore, we showed the *P* values for multinomial regressions adjusted for age, education and company size, using the category of lower consumption (less than 1 time/week) as reference. 

The dairy group included whole and skim milk, yogurt, and cheese. Men consume more frequently (4 times per week or more) whole milk while more than half of woman (54.5%) has a consumption of less than 1 time per week. Skim milk and yogurt are rarely consumed by workers, but the weekly consumption was more frequently among woman. The cheese daily consumption was more frequently among woman (19.5% versus 10.4%  *P* ≤ 0.001) than in men. 

Bread, rice, and pasta constituted the cereals group. Bread and rice was widely consumed by workers being more frequently consumed among men. In relation to pasta consumption, the weekly consume was the most frequently among workers and the daily consumption was more frequent among men (21.7% versus 11.9%  *P* ≤ 0.001). In relation to fruit and vegetables, woman presents a higher daily consumption of fruit, vegetables, and leafy vegetables while men consumed these items weekly. Beans also were widely consumed among men and women being the daily consumption higher among men. Sweets were daily consumed by 23.6% of men and 16.2 of woman (*P* ≤ 0.001). 

The meat group included sausage, fish, red meat, and chicken with and without skin. Sausages and fish were more frequently consumed weekly and by men. The frequently red meat consumption (at least 4 times per week) was higher among men, though the weekly consumption was more frequently between woman. Chicken without skin were more consumed by woman while chicken with skin were more consumed by men. The food items of oil and fat group were rarely consumed between men and woman, except bread with butter and margarine that shows a daily intake of at least more than 30% of workers. The consumption of food with lard and bacon had no association in the adjusted analysis. 

Fast food group had a lower consumption between men and woman mainly cheeseburger and pizza. More than 60% of male workers and 40% of female workers consumed soda at least one time per week. The consumption of pizza had no association with the multinomial regression. 

## 4. Discussion

This was the first nationwide description of food items consumed by the workforce in Brazil. The pattern of consumption was presented by multidimensional graphics, which permit to have an overall view of distribution of food items consumed just by visual inspection. The multidimensional graphics allow noticing differences between of dietary habits by education and sex. This graphical approach describing food intake has been used by the European Prospective Investigation into Cancer and Nutrition (EPIC) to compare the average of food intake among countries [21]. Graphics were created for food items consumed daily and rarely or never, since these extreme categories provide meaningful information regarding eating habits. 

Therefore, some graphics did not show differences based on visual inspection, since there are lines overlapping. We focused the analysis on those lines which did not overlap. The analyses identified differences in food items consumed across sex, age and education level. Moreover, the analyses using the Pearson chi-square and the multinomial regression made possible to identify independent associations of food intake by sex and the associations were adjusted for socioeconomic and demographic characteristics. Previous studies have described the association between individual characteristics and dietary patterns [8, 9]. In the present analysis, we included the size of the company as part of the explanatory equation, since the data collection has been done by the region of the country, which is an important explanatory variable. 

Overall, the eating habits of industry workers were constituted mainly by daily intake of rice, beans, bread, vegetables and green leafy vegetables, as it has been reported in surveys of woman living in southern Brazil [8] and urban Brazilian adults, aged 20 to 50 years, from the northeast and Southeast regions [9]. A study describing dietary patterns of European countries [21] identified differences by countries and sex. The Spanish dietary pattern was characterized by high intake of legumes, vegetable oils, fruits, vegetables and animal food groups, particularly seafood, egg, and milk; while in Italy and Greece dietary patterns were mostly constituted by a composite of plant-based Mediterranean food. In our study, the daily intake of red meat was reported by less than a third of workers and beans and rice dominated the food pattern independently of sex, educational level, and company size. The multidimensional graphics for men and women showed that daily intake of vegetables, fruits and dairy products had similar shapes, but with larger areas for women. In the opposite side, the daily intake of beans and rice, pasta and bread, and fruit juice had greater areas for men. These findings were confirmed in the multivariate analysis, taking into account confounding factors. 

There were differences in dietary patterns across age categories, with similar type of food items, but different magnitudes of daily consumption. Workers aged 50 years old or older tended to have greater frequency of daily intake than those 15–29 years old, particularly for vegetables, green leafy vegetables and fruits. Low education level and young age have been detected as risk factors for unhealthy diet [22]. The mechanism by which high education level promotes healthy diets have not been fully elucidated, but might be related to the exposure to knowledge, experiences, attitudes and beliefs associated with more years of formal education [23]. This study corroborated the finding of socioeconomic status as a determinant of eating habits, which might affect health promotion programs at the worksite [24]. 

The graphics presentation allowed describing the frequency of workers who already follow the frequency of intake of fruits, vegetables, and dairy products recommended by the World Health Organization [25] and the Dietary Guide for Americans [26]. On the other side, food items rarely or never consumed provide insight about the gap to be fulfilled in health interventions. In this study less than 50% of worker had a daily intake of dairy products, confirming the findings observed among workers of metal industry in Rio de Janeiro, Brazil [16]. Male sex was identified as a risk factor for inadequate intake of skim milk and female sex for milk and dairy products. 

In Brazil, the consumption of some kinds of meat varies by regions of the country. For instance, barbecue (“churrasco”) is regularly consumed in all states, while salted meat “carne de sol” is most often consumed in the northeast. However, less than 30% of workers had a daily intake of red meat, which might be explained by the domain of other categories between rarely or never and daily consumption. 

The food items with high carbohydrate or fat content were not consumed daily or rarely. However, it does not reduce their impact on prevalence of overweight and obesity [27, 28] since the intake was not measured quantitatively and it was based on frequency rather than amount. 

The main limitation of our investigation is that the food frequency questionnaire included only 46 food items. However, there were food items typical of each region of the country. The strength of our study is the large and representative sample of workers of the Brazilian industries. 

 In conclusion, we were able to describe food items daily and rarely consumed by Brazilian workers through graphic and analytical approaches. We demonstrated that the consumption of healthier foods, particularly dairy products, vegetables, and fruits, is insufficient. This deficit is remarkable among younger and less educated workers and it was not influenced by sex and size of the companies. The influence of age and sex over dietary patterns has important implications in terms of worksite health promotion interventions since it is clear that targeted approaches are needed for men, women, and different age groups. The food items consumed daily might be used to make specific recommendations in order to improve the dietary habits of workers who never consume healthy food. This full-screen image of eating habits of the Brazilian labor force can be used for planning interventions aimed at improving the quality of diets in the workplace.

## Figures and Tables

**Table 1:** Characteristics of the Brazilian workers of the SESI study ((%) or mean ± standard deviation).

	Male* (*N* = 3686)	Female* (*N* = 1132)	Total* (*N* = 4818)
Age (years)			
15–29	33.3	40.4	35.0
30–39	29.1	31.6	29.7
40–49	25.2	22.6	24.6
50–76	12.3	5.4	10.7
Education (years)			
0–4	20.6	11.9	18.6
5–8	33.7	25.6	31.8
9–11	32.3	35.5	33.0
≥12	13.4	26.9	16.6
Size of company			
Small	32.6	35.4	33.2
Medium	34.5	34.6	34.6
Large	32.9	30.0	32.2
Body mass index (kg/m^2^)			
<18.5	1.6	2.1	1.7
18.5–24.9	47.1	52.7	48.9
25.0–29.9	39.7	31.8	37.9
≥30.0	11.6	13.3	12.0
Systolic blood pressure (mmHg)	130.9 ± 18.3	114.3 ± 17.5	127.0 ± 19.5
Diastolic blood pressure (mmHg)	78.0 ± 12.3	71.4 ± 11.2	76.5 ± 12.4

^∗^Percentages were obtained in the analysis taking into account the sampling design.

**Table 2:** Food frequency consumption of cereals products and dairy products (*N* (%)) and adjusted multinomial regression, SESI study.

		Daily	4–6 times/week	2-3 times/week	1 time/week	Less than 1 time/week (reference)	*P* value multinomial regression^##^
Dairy products	Whole milk						≤0.001
	Male	490 (13.3)*	858 (23.3)*	338 (9.2)*	348 (9.4)*	1652 (44.8)	
	Female	97 (8.6)	246 (21.7)	79 (7.0)	93 (8.2)	617 (54.5)	
	Skim milk						≤0.001
	Male	80 (2.2)*	149 (4.0)*	104 (2.8)	32 (0.9)	3322 (90.1)	
	Female	76 (6.7)	72 (6.4)	41 (3.6)	11 (1.0)	931 (82.3)	
	Yogurt						≤0.001
	Male	92 (2.5)*	68 (1.8)*	376 (10.2)*	309 (8.4)*	2837 (77.1)	
	Female	102 (9.0)	37 (3.3)	147 (13.0)	146 (12.9)	699 (61.8)	
	Cheese						≤0.001
	Male	382 (10.4)*	229 (6.2)	842 (22.8)	548 (14.9)	1684 (45.7)	
	Female	220 (19.5)	82 (7.3)	228 (20.2)	175 (15.5)	425 (37.6)	

Cereals products	Bread						0.05
	Male	2845 (77.2)^#^	310 (8.4)	229 (6.2)	182 (4.9)	120 (3.3)	
	Female	760 (67.1)	107 (9.5)	129 (11.4)	96 (8.5)	40 (3.5)	
	Rice						≤0.001
	Male	3377 (91.7)*	168 (4.6)	70 (1.9)	19 (0.5)	49 (1.3)	
	Female	934 (82.6)	94 (8.3)	56 (5.0)	16 (1.4)	31 (2.7)	
	Pasta						≤0.001
	Male	800 (21.7)*	338 (9.2)*	1371 (37.2)*	694 (18.8)	480 (13.0)	
	Female	134 (11.9)	101 (8.9)	427 (37.8)	283 (25.1)	184 (16.3)	

Fruits and vegetables	Fruit juice						≤0.001
	Male	1357 (36.8)*	366 (9.9)*	518 (14.1)*	507 (13.8)	938 (25.4)	
	Female	348 (30.8)	92 (8.1)	152 (13.4)	212 (18.7)	327 (28.9)	
	Fruit						≤0.001
	Male	1017 (27.6)*	389 (10.6)	1195 (32.5)*	453 (12.3)	626 (17.0)	
	Female	467 (41.3)	115 (10.2)	263 (23.3)	122 (10.8)	164 (14.5)	
	Vegetables						≤0.001
	Male	1831 (49.7)	524 (14.2)*	842 (22.9)*	252 (6.8)	234 (6.4)	
	Female	642 (56.8)	134 (11.8)	210 (18.6)	69 (6.1)	76 (6.7)	
	Leafy vegetables						≤0.001
	Male	1597 (43.3)*	454 (12.3)	867 (23.5)*	330 (9.0)	437 (11.9)	
	Female	606 (53.6)	134 (11.9)	187 (16.6)	83 (7.4)	119 (10.5)	

Beans	Beans						≤0.001
	Male	3166 (86.1)*	227 (6.2)*	172 (4.7)	43 (1.2)	68 (1.8)	
	Female	735 (65.0)	82 (7.3)	161 (14.2)	65 (5.7)	88 (7.8)	
Sweets	Sweets						≤0.001
	Male	596 (16.2)*	277 (7.5)*	764 (20.8)	521 (14.2)	1519 (41.3)	
	Female	266 (23.6)	114 (10.1)	187 (16.6)	173 (15.3)	389 (34.5)	

Meats	Sausages						0.03
	Male	123 (3.3)	161 (4.4)	951 (25.8)*	909 (24.7)	1540 (41.8)	
	Female	36 (3.2)	45 (4.0)	253 (22.4)	276 (24.4)	520 (46.0)	
	Fish						≤0.001
	Male	55 (1.5)	114 (3.1)	691 (18.8)*	1005 (27.3)*	1818 (49.4)	
	Female	13 (1.1)	27 (2.4)	152 (13.4)	273 (24.1)	666 (58.9)	
	Red meat						≤0.001
	Male	902 (24.5)*	778 (21.1)*	1503 (40.8)*	327 (8.9)	176 (4.8)	
	Female	189 (16.7)	205 (18.1)	479 (42.4)	154 (13.6)	103 (9.1)	
	Chicken with skin						≤0.001
	Male	92 (2.5)	284 (7.7)*	1018 (27.7)*	461 (12.6)*	1816 (49.5)	
	Female	35 (3.1)	66 (5.9)	203 (18.2)	136 (12.2)	677 (60.6)	
	Chicken without skin						≤0.001
	Male	191 (5.2)*	390 (10.6)*	1199 (32.5)*	479 (13.0)*	1425 (38.7)	
	Female	75 (6.7)	181 (16.2)	391 (34.9)	160 (14.3)	312 (27.9)	

Oils and fats	French fries						≤0.001
	Male	84 (2.3)	114 (3.1)	772 (21.0)*	827 (22.5)*	1882 (51.2)	
	Female	19 (1.7)	28 (2.5)	196 (17.3)	248 (21.9)	639 (56.5)	
	Fried food						≤0.001
	Male	132 (3.6)	197 (5.4)*	753 (20.5)*	633 (17.2)	1965 (53.4)	
	Female	32 (2.8)	40 (3.5)	196 (17.3)	233 (20.6)	630 (55.7)	
	Bread with margarine						≤0.001
	Male	1677 (45.5)	180 (4.9)	128 (3.5)^#^	81 (2.2)*	1621 (44.0)	
	Female	465 (41.1)	56 (4.9)	55 (4.9)	55 (4.9)	501 (44.3)	
	Bread with butter						≤0.001
	Male	1173 (31.8)*	134 (3.6)	93 (2.5)*	82 (2.2)	2204 (59.8)	
	Female	245 (21.7)	43 (3.8)	62 (5.5)	26 (2.3)	755 (66.8)	
	Food with lard						0.2
	Male	114 (3.1)	28 (0.8)	80 (2.2)	57 (1.5)	3404 (92.4)	
	Female	38 (3.4)	15 (1.3)	20 (1.8)	23 (2.0)	1036 (91.5)	
	Bacon						0.1
	Male	35 (1.0)	45 (1.2)	217 (5.9)	329 (8.9)	3054 (83.0)	
	Female	8 (0.7)	28 (2.5)	58 (5.1)	87 (7.7)	949 (84.0)	
Fast food	Soda						≤0.001
	Male	562 (15.3)*	516 (14.0)*	1179 (32.0)*	615 (16.7)*	810 (22.0)	
	Female	111 (9.8)	84 (7.4)	277 (24.5)	252 (22.3)	406 (35.9)	
	Bread with mayonnaise						≤0.001
	Male	615 (16.7)	40 (1.1)	30 (0.8)*	25 (0.7)	2976 (80.7)	
	Female	180 (15.9)	17 (1.5)	34 (3.0)	12 (1.1)	888 (78.5)	
	X burger						0.003
	Male	25 (0.7)^#^	27 (0.7)	258 (7.0)*	486 (13.2)^#^	2888 (78.4)	
	Female	3 (0.3)	11 (1.0)	61 (5.4)	156 (13.8)	899 (79.6)	
	Pizza						0.7
	Male	19 (0.5)	27 (0.7)	224 (6.1)	701 (19.0)	2711 (73.6)	
	Female	4 (0.4)	12 (1.1)	93 (8.2)	250 (22.1)	771 (68.2)	

*P* values of chi-square test: *P value <0.001. ** 0.01 ≤ *P* value ≤0.049. ^##^ *P* value adjusted for age, education, size of company.
